# Clinical and Economic Impact of Previous Bariatric Surgery on Liver Transplantation: a Nationwide, Population-Based Retrospective Study

**DOI:** 10.1007/s11695-021-05684-4

**Published:** 2021-09-09

**Authors:** Antonio Iannelli, Julie Bulsei, Tarek Debs, Albert Tran, Andrea Lazzati, Jean Gugenheim, Rodolphe Anty, Niccolo Petrucciani, Eric Fontas

**Affiliations:** 1 Université Côte D’Azur, Nice, France; 2 Centre Hospitalier Universitaire de Nice - Digestive Surgery and Liver Transplantation Unit, Archet 2 Hospital, 151 Route Saint Antoine de Ginestière, BP 3079 Nice Cedex 3, France; 3 Inserm, U1065, Team 8 “Hepatic Complications of Obesity and Alcohol”, Nice, France; 4 Centre Hospitalier Universitaire de Nice, Department of Clinical Research and Innovation, Université Côte D’Azur, Nice, France; 5 Department of General Surgery, Centre Hospitalier Intercommunal de Creteil, Creteil, France; 6 Department of Medical and Surgical Sciences and Translational Medicine, Faculty of Medicine and Psychology, St. Andrea Hospital, Sapienza University, Rome, Italy

**Keywords:** Liver transplantation, Cost, Mortality, Re-transplantation, Obesity

## Abstract

**Purpose:**

The present study aims to determine the impact of previous bariatric surgery (BS) on the length of hospital stay; the incidence of mortality, re-transplantation, and re-hospitalization after LT; and the related economic costs, through the analysis of the French National Health Insurance Information System.

**Materials and Methods:**

All patients aged > 18 years who underwent LT in France in the period from 2010 to 2019 were included. Thirty-nine patients with a history of BS (study group) were compared with 1798 obese patients without previous BS (control group).

**Results:**

At the time of LT, patients with a history of BS were significantly younger than those of the control group and had lower Charlson comorbidity index. Female sex was significantly more represented in the study group. No significant differences were detected between the two groups regarding the postoperative mortality rate after LT (10.3% in the study group versus 8.0% in the control group), long-term mortality (0.038 versus 0.029 person-year of follow-up, respectively), re-transplantation (adjusted hazard ratio (*HR*) = 2.15, *p* = 0.2437), re-hospitalization (adjusted analysis, *IRR* = 0.93, *p* = 0.7517), and costs of LT hospitalization (73,515 € in the study group versus 65,878 € in the control group). After 1:2 propensity score matching, the duration of the LT hospital stay was significantly longer in the study group (58.3 versus 33.4 days, *p* = 0.0172).

**Conclusion:**

No significant differences were detected between patients with previous BS versus obese patients without history of BS undergoing LT concerning the rates of mortality, re-LT, re-hospitalization after LT, and costs of hospitalization and re-hospitalizations.

**Graphical abstract:**

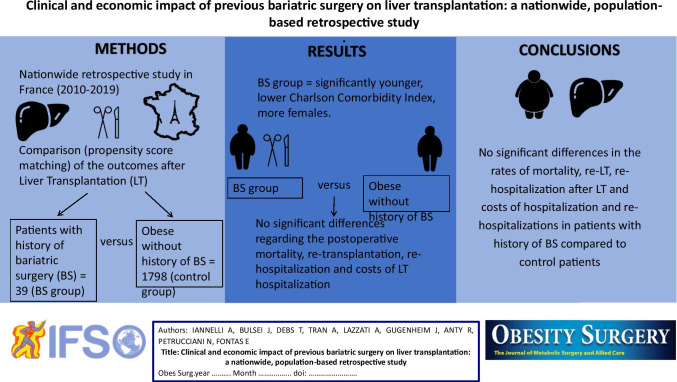

**Supplementary Information:**

The online version contains supplementary material available at 10.1007/s11695-021-05684-4.

## Introduction

The number of individuals with obesity undergoing bariatric surgery (BS) has sharply increased in the last two decades in concomitance with the epidemic of obesity and the wide diffusion of BS [1–3]. While BS has been shown to have a protective effect against the onset and/or the progression of non-alcoholic steatohepatitis (NASH) and liver cirrhosis [4], patients with a previous history of BS may become candidate to liver transplantation (LT) because of acute or chronic liver failure or development of primary liver tumors [5]. Alcohol-related end-stage liver disease may occur due to a shift in the addictive profile from food to alcohol [6–8], the occurrence of hepatocarcinoma (HCC) on NASH, or the progression of NASH in spite of BS [9, 10]. Whatever the indication for LT, liver transplant surgeons are facing more and more patients candidates to LT with a previous history of BS, and it may be speculated that in the foreseeable future, this association will be even more frequent due to the deep penetration of BS and the worldwide epidemic of obesity [11–13]. Furthermore, BS is being used to favor access to LT in patients with obesity that would otherwise not be listed because LT is challenging in this category of patients and several transplant centers have fixed a body mass index (BMI) cutoff at 35 kg/m^2^ or 40 kg/m^2^ to get access to LT program [14].

A few monocentric series have reported the results of LT in patients with a history of BS, including a small number of patients in each study [7, 15–18]. The majority of single-center studies did not find significant differences in the outcomes of LT between patients with or without history of BS [7, 15, 18]. On the other hand, Idriss et al. [17] reported a higher rate of delisting for LT and lower survival from the time of listing in cirrhotic patients with a history of BS compared to patients with no history of BS. However, survival from the time of LT was similar between patients with or without previous BS [17]. The most recent systematic review and meta-analysis on this topic identified a total of 187 patients in 8 studies who underwent BS before LT [19]. Thus, there is an urgent need to share the results of patients undergoing both BS and LT in different sequences, giving the scarcity of data worldwide.

The present study aims to determine the impact of previous history of BS on the length of hospital stay for LT, the incidence of mortality, re-transplantation and re-hospitalization after LT, and the related economic costs, through the analysis of the French National Health Insurance Information System between 2010 and 2019.

We hypothesized that BS might reduce patients’ comorbidities and consequently the mortality rate, length of stay, and LT-related costs as compared to patients with obesity and no history of BS.

## Materials and Methods

### Study Design

The present study is an observational descriptive study that compared outcomes during LT hospitalization and follow-up between patients who underwent LT with a previous history of BS (study group) and patients with a diagnosis of obesity who underwent LT without a previous history of BS (control group). The institutional review committee approved the study. Data were extracted from the French national hospital discharge database (“Programme De Médicalisation des Systèmes d’Information,” PMSI), which is used for billing hospitalizations in all French hospitals, irrespective of their academic affiliation or ownership (public and private for-profit and private non-profit). Because discharge reports are mandatory and constitute the basis of hospital funding, this database is exhaustive on all reimbursed hospital stay, including surgical interventions, in the country.

In the PMSI database, data are collected as standardized discharge reports, consisting of patient demographic data (age, gender, zip code, entry, and release dates); primary and associated diagnoses based on the International Classification of Disease, 10th edition (ICD-10); and therapeutic procedures based on the Common Classification of Medical Acts (Classification Commune des Actes Médicaux, CCAM, 11th edition), which is a national standardized classification of medical procedures [20]. Each patient in the database is identified with a unique anonymous identifier, which allows for linkages between consecutive hospital stays in different hospitals. Since the individual information is anonymous and publicly available, patient consent is not required.

We included all patients aged > 18 years who underwent LT in France in the period from January 1, 2010, to December 31, 2019 (ICD-10, Z94-4). Follow-up ended on the December 31, 2019, or the date of death, or in case of re-transplantation, the date of the end of the hospitalization for re-transplantation, whichever occurred first. We selected patients with an obesity diagnosis (ICD-10*,* E66x) at the BS hospitalization in the study group and during the year before LT in the control group. BS, selected in the 2010–2019 period, was identified using the following codes: open adjustable gastric banding (AGB) (CCAM, HFMA009, HFMA006, HFKA002); laparoscopic AGB (CCAM, HFMC007, HFMC005, HFKC001); open Roux-en-Y gastric bypass (RYGB) (CCAM, HFCA001, HGCA009, HFFA001); laparoscopic RYGB (CCAM, HFCC003, HGCC027, HFFC004); open sleeve gastrectomy (SG) (CCAM, HFMA010, HFFA011); and laparoscopic SG (CCAM, HFMC006, HFFC018). Exclusion criteria were patients receiving synchronous BS and LT and patients receiving BS after LT.

The analyzed covariates were demographic, age, gender, and body mass index (BMI) (stratified as follows: BMI from 30 to 39.9 kg/m^2^, from 40 to 49.9 kg/m^2^, and ≥ 50 kg/m^2^); the severity grade, a score provided by the PMSI database referring to the complexity of the hospitalization [21]; obesity-related comorbidities, hypertension (ICD-10, I10), diabetes (ICD-10, E10-X – E14-X), obstructive sleep apnea syndrome (OSAS) (ICD-10, G47-3), hypercholesterolemia (ICD-10, E78-0), hyperlipidemia, or use of lipid-lowering agents (ICD-10, ET8-5); Charlson comorbidity index (CCI); and etiology of liver disease, non-alcoholic steatohepatitis (NASH) (ICD-10, K74-6, K75-8), hepatitis B virus (HBV) (B19.10, B18.1), hepatitis C virus (HCV) (ICD-10, B18-2), alcoholic liver disease (ALD) (ICD-10, K70-3), acute or fulminant liver failure (ICD-10, K71-2, K71-1), and hepatocellular carcinoma (HCC) (ICD-10, C22.0).

### Primary Outcomes

The primary outcomes of interest were LT postoperative in-hospital mortality (defined as mortality during the LT hospitalization); incidence of re-transplantation (ICD-10, Z94-4); and death and re-hospitalization during follow-up.

### Secondary Outcomes

The secondary outcomes were length of LT hospital stay and intensive care unit (ICU) stay (days); costs related to the LT hospitalization (euros); and costs related to re-hospitalizations (euros).

### Statistical Analysis

Patients’ characteristics in the two groups were described by mean (standard deviation) for quantitative data and frequency (percentage) for qualitative data and compared using, respectively, the Student’s *t* test and the Chi^2^ test or Fisher’s exact test.

Due to the different duration of follow-up for each patient, the incidence of re-LT, deaths, and re-hospitalizations was expressed as the number of new cases per person-year of follow-up (PYFU), and the costs of re-hospitalizations were expressed in euros (€) per PYFU.

We studied the associations between BS before LT and our outcomes using univariate models secondarily adjusted on age, sex, and CCI. Mortality during LT hospitalization was studied through logistic regression models. Re-LT and death were studied using Cox models and re-hospitalizations using Poisson regression models. The length of LT hospital stay and ICU stay was analyzed using linear regression models.

Costs were compared using generalized linear regression with gamma distribution and log link. Costs of hospitalization were estimated from the payer’s perspective, using the reimbursement values defined by the National Health Insurance (Assurance Maladie), including health workers salaries, medical costs (drugs, equipment, interventions), hospitalization costs (food, heating), and management costs, and were reported in euros 2019.

In order to assess the uncertainty around the results, a 1:2 propensity score matching was performed as a sensitivity analysis using age, sex, and CCI as matching variables to analyze the variables described above.

All tests were two-sided and *p* values < 0.05 were considered statistically significant. We used SAS Enterprise Guide software version 7.1 (SAS institute, Inc., Cary, North Carolina, USA) for statistical analyses.

## Results

Thirty-nine patients who had LT after BS (study group) and 1798 patients with obesity without history of BS who underwent LT (control group) between 2010 and 2019 were identified and constitute the basis of the present study. The mean duration of follow-up was 2.03 years ± 2.1 for the 39 patients who underwent LT after BS (study group) versus 3.4 ± 2.6 years in the control group (*p* = 0.0011).

### Patients’ Characteristics at the Time of BS and LT

The characteristics of the study group and control group are reported in Table 1.

**Table 1:** Patients’ characteristics in the study group (patients with previous history of bariatric surgery who underwent liver transplantation) and control group (patients with a diagnosis of obesity who underwent liver transplantation)

	Study group ***N*** = 39	Control group ***N*** = 1798	***p*** value
Patients characteristics at the time of LT			
Age (years), mean (SD)	47.3 (10.8)	57.6 (8.2)	< 0.0001^a^
Sex, frequency (%)			
Female	25 (64.1)	409 (22.7)	< 0.0001^b^
Male	14 (35.9)	1389 (77.3)	
Charlson comorbidity index (not weighted by age), mean (SD)	3.8 (1.9)	5.1 (1.3)	0.0003^a^
Hospitalization for LT			
Severity grade, frequency (%)			
Grade 1	0 (0.0)	42 (2.3)	0.5639^c^
Grade 2	4 (10.3)	305 (17.0)	
Grade 3	12 (30.8)	585 (32.5)	
Grade 4	23 (59.0)	866 (48.2)	
Surgical procedure, frequency (%)			
Split liver transplantation	0 (0.0)	37^1^ (2.0)	1.0000^c^
Total liver transplantation	40^1^ (100.0)	1765^1^ (98.0)	
Main diagnosis, frequency (%)			
Acute liver failure	7 (18.0)	70 (3.9)	< 0.0001^c^
Alcoholic cirrhosis	6 (15.4)	411 (22.9)	
Cirrhosis, not otherwise specified	4 (10.3)	162 (9.0)	
Hepatocellular carcinoma	2 (5.1)	527 (29.3)	
Other	20 (51.3)	628 (34.9)	
Other associated diagnoses, frequency (%)			
Alcoholic cirrhosis	8 (20.5)	737 (41.0)	< 0.0001^c^
Cirrhosis, not otherwise specified	10 (25.6)	593 (33.0)	
Arterial hypertension	12 (30.8)	867 (48.2)	
Chronic viral hepatitis C	1 (2.6)	209 (11.6)	
Chronic viral hepatitis B	0 (0.0)	83 (4.6)	
Liver toxic disease with acute hepatitis	0 (0.0)	1 (0.1)	
Type 1 diabetes	2 (5.1)	202 (11.2)	
Diabetes, not otherwise specified	0 (0.0)	35 (1.9)	
Obstructive apnea syndrome	1 (2.6)	134 (7.5)	
Hypercholesterolemia	0 (0.0)	77 (4.3)	
Hyperlipidemia	0 (0.0)	53 (2.9)	
Diagnosis of obesity, frequency (%)	9 (23.1)	1798 (100.0)	
Availability of BMI, frequency (%)	6 (15.4)	1068 (59.4)	
Hospitalization for BS			
Surgical procedure, frequency (%)		NA	NA
RYGB	21 (53.9)		
Sleeve gastrectomy	14 (35.9)		
Vertical banded gastroplasty	1 (2.6)		
Adjustable gastric banding	3 (7.7)		
BMI class, frequency (%)			
30 to 40 kg/m^2^	11 (28.2)		
40 to 50 kg/m^2^	25 (64.1)		
> 50 kg/m^2^	3 (7.7)		
Years between BS and LT, mean (SD)	3.4 (2.3)		

^1^Some patients received liver re-transplantation during the same hospital stay

^a^Student’s *t* test

^b^Chi^2^ test

^c^Fisher’s exact test

*N*, number of patients; *LT*, liver transplantation; *SD*, standard deviation; *BMI*, body mass index; *BS*, bariatric surgery; *RYGB*, Roux-en-Y gastric bypass

The most frequent bariatric surgical procedure was RYGB, in 21 (53.9%) patients, followed by SG in 14 (35.9%) cases. The majority of patients had a BMI ranging from 40 to 50 kg/m^2^ at the time of BS. The mean time frame between BS and LT was 3.4 years.

At the time of LT, patients with a history of BS were significantly younger than those of the control group and had lower CCI (Table 1). Female sex was significantly more represented in the study group. No significant difference was detected in the severity grade of the hospitalization for LT or in the type of LT (split versus total LT). Significant differences were detected in the main and associated diagnoses, with a higher rate of HCC in the control group and of acute liver failure in the study group. Arterial hypertension, obstructive sleep apnea syndrome, and chronic viral C hepatitis were more frequent in the control group. BS was effective in inducing weight loss as the diagnosis of obesity was coded in only 23.1% of patients in the study group.

### Primary Outcomes

During the hospital stay for LT, four deaths in the study group (10.3%) and 144 deaths (8.0%) in the control group were reported, and no association was found between the group and mortality (adjusted odds ratio (*OR*) = 1.14, 95% confidence interval (CI) [0.38; 3.39], *p* = 0.8159).

During follow-up, mortality was 0.038/ PYFU in the study group versus 0.029/PYFU in the control group. No association was found between the groups and occurrence of re-transplantation (adjusted hazard ratio (*HR*) = 2.15, 95% CI [0.59; 7.79], *p* = 0.2437), death (adjusted *HR* = 1.69, 95% CI [0.53; 5.44], *p* = 0.3759), and re-hospitalization (adjusted analysis, *IRR* = 0.93, 95% CI [0.60; 1.44], *p* = 0.7517) (Table 2).

**Table 2:** Comparison of long-term outcomes after LT in the study group (patients with previous history of bariatric surgery who underwent liver transplantation) and control group (patients with a diagnosis of obesity who underwent liver transplantation)

	Event incidence per PYFU [95% CI]		Models analysis			
	Study group ***N*** = 39	Control group ***N*** = 1798	Univariate analysis^a^		Multivariate analysis^b^	
			HR [95 CI]	***p*** value	HR [95 CI]	***p*** value
Re-LT	0.038 [0.000; 0.081]	0.007 [0.005; 0.009]	4.75 [1.47; 15.36]	0.0093	2.15 [0.59; 7.79]	0.2437
Death	0.038 [0.000; 0.081]	0.029 [0.024; 0.033]	1.24 [0.40; 3.89]	0.7121	1.69 [0.53; 5.44]	0.3759
			Univariate analysis^c^		Multivariate analysis^d^	
			IRR [95 CI]	***p*** value	IRR [95 CI]	***p*** value
Re-hospitalizations	2.9 [2.5; 3.3]	2.4 [2.3; 2.4]	0.94 [ 0.60; 1.47]	0.7945	0.93 [0.60; 1.44]	0.7517

^a^Cox model, unadjusted

^b^Cox model adjusted for age, sex, and CCI

^c^Poisson regression model, unadjusted

^d^Poisson regression model adjusted for age, sex, and CCI

*N*, number of patients; *LT*, liver transplantation; *SD*, standard deviation; *CI*, confidence intervals; *CCI*, Charlson comorbidity index; *PYFU*, person-year of follow-up; *HR*, hazard ratio; *IRR*, incidence rate ratio

### Secondary Outcomes

No significant difference was found between the groups regarding LT hospital stay and intensive care unit stay (Table 3). The costs of LT hospitalization were 73,515€ in the study group versus 65,878€ in the control group. The costs of re-hospitalizations were 13,484€/PYFU in the study group versus 7745 in the control group. Comparison of costs of the LT hospitalization and of re-hospitalizations between the study and the control group did not show any significant difference, as reported in Table 4.

**Table 3:** Comparison of hospital and intensive care unit (ICU) stay after LT in the study group (patients with previous history of bariatric surgery who underwent liver transplantation) and control group (patients with a diagnosis of obesity who underwent liver transplantation)

	Study group ***N*** = 39	Control group ***N*** = 1798	Univariate analysis^a^		Multivariate analysis^b^	
			β [95% CI]	***p*** value	β [95% CI]	***p*** value
Hospital stay (days), mean (SD)	58.28 (71.23)	42.71 (42.54)	0.22 [− 0.03; 0.46]	0.0789	0.18 [− 0.06; 0.42]	0.1493
ICU stay (days), mean (SD)	17.69 (23.63)	13.78 (23.36)	0.29 [− 0.09; 0.67]	0.1401	0.21 [− 0.18; 0.60]	0.2926

^a^Univariate linear regression with logarithmic transformation (natural logarithm) of the duration of the hospital stay and ICU stay

^b^Linear multivariate regression adjusted for age, sex, and CCI, with logarithmic transformation (natural logarithm) of the duration of the hospital stay and ICU stay

*N*, number of patients; *LT*, liver transplantation; *SD*, standard deviation; *ICU*, intensive care unit; *CI*, confidence intervals, *CCI*, Charlson comorbidity index

**Table 4:** Comparison of costs after LT in the study group (patients with previous history of bariatric surgery who underwent liver transplantation) and control group (patients with a diagnosis of obesity who underwent liver transplantation)

	Study group ***N*** = 39	Control group ***N*** = 1798	Univariate analysis^a^		Multivariate analysis^b^	
			β [95 CI]	***p*** value	β [95 CI]	***p*** value
Costs of hospitalization for LT (€), mean (SD)	73,515 (50,188)	65,878 (39,770)	0.11 [− 0.03; 0.25]	0.1262	0.06 [− 0.09; 0.20]	0.4358
			Univariate analysis^c^		Multivariate analysis^d^	
			β [95 CI]	***p*** value	β[95 CI]	***p ***value
**Costs of re-hospitalization (€)**, for PY of follow-up [95% CI]	13,484 [13459; 13509]	7745 [7743; 7748]	0.15 [− 0.53; 0.84]	0.6537	0.23 [− 0.49; 0.95]	0.5175

^a^Generalized linear regression with gamma distribution and log link

^b^Generalized linear regression with gamma distribution and log link adjusted for age, sex, and CCI

^c^Generalized linear regression with gamma distribution and log link

^d^Generalized linear regression with gamma distribution and log link adjusted for age, sex, and CCI

*N*, number of patients; *LT*, liver transplantation; *SD*, standard deviation; *CI*, confidence intervals; *CCI*, Charlson comorbidity index; *PY*, person-year; *€*, euros

### Sensitivity Analysis and Propensity Score Matching

This analysis included 39 patients in the study group with a matched control group of 78 patients (Supplementary Tables 5 and 6). After matching, our results were in accordance with the previously described models analyses showing no significant differences in the occurrence of re-transplantation, deaths, re-hospitalization, length of ICU stay, and costs. However, the duration of the LT hospital stay was significantly longer in the study group (58.3 versus 33.4 days in the matched control group, *p* = 0.0172).

## Discussion

This study provides evidence that (1) previous BS does not affect outcomes (mortality, re-LT, re-hospitalization) after LT; (2) patients with history of BS represent a peculiar population with distinct characteristics, including higher prevalence of women, younger age, lower CCI, and different distribution of the indications for LT compared to patients with obesity who undergo LT without history of BS; and (3) postoperative outcomes and costs of hospitalization and re-hospitalization are similar in patients with or without previous BS.

Our findings are relevant as they provide interesting additional information on an emerging topic, LT in individuals with obesity and a history of BS. The number of patients with history of BS needing LT is expected to rise in the next decades, due to the epidemic of obesity, the subsequent augmentation of obesity-related liver diseases, and the large diffusion of BS. Obesity is associated with the occurrence of several comorbidities including non-alcoholic fatty liver disease (NAFLD) and non-alcoholic steatohepatitis (NASH), representing the fastest growing indication for LT in Western countries [22, 23]. Furthermore, other indications for LT in the subset of patients with history of BS may exist or coexist including HCC, viral hepatitis, alcohol-related diseases, biliary diseases, and other causes. Acute liver failure due to protein malnutrition and bacterial overgrowth after BS remains a rare indication for LT [24].

In the present study, we found a significantly higher rate of female patients, a lower mean age, and a lower CCI in the group of patients with a history of BS. The higher percentage of women is certainly related to the gender disparities in seeking and receiving BS [25]. It is estimated that approximately 70–80% of patients undergoing BS are women [25, 26]. In this series, women constituted 64.1% of the study group versus only 22.7% of the control group. The group of patients undergoing LT after BS also had a lower CCI and lower mean age (with a 10-year difference in the means). These results are similar to those recently reported by Fipps et al., showing higher rate of female sex and younger age in the cohort who had previous BS [8]. We found several differences also in the indications for LT. The main reason for LT was hepatocellular carcinoma (HCC) in the group of obese patients without history of BS, accounting for 29.3% of the indications, whereas only 5.1% of patients in the study group were transplanted for HCC. On the other hand, acute liver failure was the indication for LT in 18% in the BS group versus only 3.9% in the control group. The higher rate of acute liver failure may be explained by the occurrence of liver complications after BS, in which protein-caloric malnutrition, bacterial overgrowth with increased intestinal permeability, lipotoxicity, and genetic background seem all to play a role [27] and by a lower tolerance to acute liver injury after BS [28]. On the other hand, the younger age of the patients in the BS group and the lower prevalence of chronic hepatitis related to viral infection may be responsible of the lower rate of HCC as an indication for LT. At the time of LT, the diagnosis of obesity was present only in 23.1% of the study group, confirming the efficacy of BS in the majority of cases. RYGB was the most frequent BS procedure, performed in 53.9% of cases, followed by SG (35.9%), which is surprising as in France SG overcame the RYGB in 2010 as the most performed procedure [2]. Furthermore, we highlight that SG may be advantageous over RYGB in this setting for the absorption of immunosuppressive drugs [29], even if close monitoring after SG to avoid possible overdosing is recommended by some authors [30].

The present study demonstrates that a history of previous BS does not affect the most relevant outcomes after LT, including in-hospital and long-term mortality, rates of re-transplantation during follow-up, and rates of re-hospitalizations. Patients with previous BS had longer mean hospital (58.3 versus 42.7 days, respectively) and ICU stay (17.7 versus 13.8) compared to the control group, even if these differences were not statistically significant. Mean costs of hospitalization and re-hospitalization were higher in the BS group, even if the difference was not statistically significant.

Statistical modeling allowed keeping all the patients of the two groups even if they showed differences in preoperative variables, as previously discussed. Although the association between the outcomes and the group has been estimated after adjusting for age, sex, and CCI, it is possible that a residual confounding bias remained. Propensity score matching was then performed to create two groups of patients with the same initial characteristics in terms of age, sex, and CCI. However, since the interest group was small (39 patients) and the matching was limited to 1 to 2, to avoid the risk of losing patients from the interest group and increasing the standardized differences, the size of the control group was drastically reduced (from 1798 patients to 79).

After propensity score matching, in accordance with the main analyses, no significant differences were detected between the two groups in the majority of outcomes, except for hospital stay, which was longer in patients with previous BS. However, although reasons for longer hospital stay are difficult to hypothesize basing on the available data, the reduced sample used in the propensity score matching may account for this difference. Indeed, when the whole population of the control group is analyzed, no difference could be demonstrated between the two groups.

While the number of patients with history of BS we found is relatively limited over a 10-year period, the present series represents the largest in the literature to date, adding significant data to the previous knowledge. Furthermore, in the present study, a large control group of 1798 patients with obesity undergoing LT in the same time frame was analyzed adding stringency to our analysis. The results of previous studies was summarized by the recent meta-analysis by Lee et al. [19], which retrieved only eight articles [15, 17, 18, 31–35] reporting results of LT after BS including a total of 187 patients. Half of the studies included patients with previous history of BS who underwent LT; the other half included patients who had BS in the effort to allow weight loss and subsequent LT. Morbidity was low after BS, with a rate of minor and major complication of 4% and 1%, respectively, at 30 days. The meta-analysis reports that 70% of the listed patients underwent LT, having a 1-year graft survival of 70%. Mortality (beyond 30 days) rate was 7% after LT, which is comparable to our results. The largest single institutional study [15] included 33 patients with a history of BS, matched to 99 without BS. The authors did not observe any deleterious effect of previous BS on the postoperative outcomes after LT. Idriss et al. [17] pointed out the need of a careful nutritional assessment in the subset of patients with a history of BS. They studied 78 patients listed for LT with a previous history of BS and compared them to a matched cohort of 156 patients. Among the 78 patients with previous BS, only 22 received LT. Bariatric surgery was associated to lower intention-to-treat survival at 1 and 3 years compared to the control group, and sarcopenia was significantly associated to delisting. However, patients with a history of BS who received LT had similar survival outcomes at 1 and 3 years of follow-up to those of the control group.

### Limits

National dataset analysis allows the inclusion of a larger study population than single institutional studies. However, data collection may be incomplete in some cases. In this study, BMI values were fully reported only at time of the hospitalization for BS. However, while at time of hospitalization for LT, BMI not reported in around half of patients, obesity was coded in 23% of patients, indicating the efficacy of BS on weight loss. Both statistical modeling and propensity score matching may be affected by a residual confounding bias, particularly related to BMI in our study. However, the propensity score analysis confirmed the results obtained with the whole population of control group mitigating the potential bias due to the incompleteness of BMI data. Due to the coding system, the diagnosis of NAFLD and NASH could not be clearly extrapolated from the diagnosis of “cirrhosis, not otherwise specified,” and “other.”

## Conclusion

Patients with previous BS undergoing LT have similar rates of mortality, re-LT, re-hospitalization after LT, and increased costs of hospitalization and re-hospitalizations compared to patients with obesity without previous BS. Further studies are encouraged to elucidate the optimal treatment of obese patients having an indication for LT.

## Supplementary Information

Below is the link to the electronic supplementary material.

Supplementary file1 (DOCX 26 KB)

## Abbreviations

CCI: Charlson comorbidity index
LT: Liver transplantation
