# Supplementary material for: Clinical and Economic Impact of Previous Bariatric Surgery on Liver Transplantation: a Nationwide, Population-Based Retrospective Study
Source: Obes Surg. 2021 Sep 9;32(1):55–63. doi: 10.1007/s11695-021-05684-4 (PMC8752569; doi:10.1007/s11695-021-05684-4)
Supplement: Supplementary file 1 — Supplementary file1 (DOCX 26 KB) [file 11695_2021_5684_MOESM1_ESM.docx]

**Table 5 (Supplementary). Patients’ characteristics after 1:2 propensity score matching**

|  | **Study group**  **N=39** | **Matched control group**  **N=78** | **P-value** |
| --- | --- | --- | --- |
| **Patients’ characteristics at the time of LT** | | | |
| **Age (years),** mean (SD) | 47.3 (10.8) | 46.8 (12.0) | 0.8311^a^ |
| **Sex,** frequency (%) |  |  |  |
| Female | 25 (64.1) | 53 (68.0) | 0.6774^b^ |
| Male | 14 (35.9) | 25 (32.0) |  |
| **Charlson Comorbidity Index (not weighted by age),** mean (SD) | 3.8 (1.9) | 4.1 (1.6) | 0.8979^c^ |

^a^ Student’s t test

^b^ Chi^2^ test

^c^ Fisher’s exact test

N, number of patients; LT, liver transplantation; SD, standard deviation

**Table 6 (Supplementary). Comparison of outcomes after LT in the study group (patients with previous history of bariatric surgery who underwent liver transplantation) and a 1:2 propensity score matched control group (patients with a diagnosis of obesity who underwent liver transplantation)**

|  | **Study group**  **N=39** | **Matched control group**  **N=78** | **Univariate analysis ^a^** | |
| --- | --- | --- | --- | --- |
|  |  |  | **β [95 CI]** | **P-value** |
| **Hospital stay (days),** mean (SD) | 58.28 (71.23) | 33.37 (20.11) | 0.33 [0.06; 0.60] | **0.0172** |
| **ICU stay (days),** mean (SD) | 17.69 (23.63) | 12.12 (12.43) | 0.22 [-0.24; 0.69] | 0.3384 |
|  |  |  | **Univariate analysis** ^b^ | |
|  |  |  | **HR [95 CI]** | **P-value** |
| **Incidence of re-LT,** for PY* of follow-up [95% CI] | 0.038 [0.000; 0.081] | 0.018 [0.005; 0.035] | 1.80 [0.40; 8.09] | 0.4410 |
|  |  |  |  |  |
| **Incidence of death,** for PY* of follow-up [95% CI] | 0.038 [0.000; 0.081] | 0.031 [0.008; 0.055] | 1.13 [0.29; 4.39] | 0.8573 |
|  |  |  | **Multivariate analysis** ^c^ | |
|  |  |  | **RR [95 CI]** | **P-value** |
| **Incidence of re-hospitalizations,** for PY* of follow-up [95% CI] | 2.9 [2.5; 3.3] | 2.4 [2.2; 2.6] | 0.99 [ 0.68 ; 1.46 ] | 0.9673 |
|  |  |  | **Univariate analysis** ^d^ | |
|  |  |  | **β [95 CI]** | **P-value** |
| **Costs of hospitalization for LT (€),** mean (SD) | 73515 (50188) | 63223 (21747) | 0.15 [-0.00; 0.30] | 0.0506 |
|  |  |  | **Multivariate analysis** ^e^ | |
|  |  |  | **β [95 CI]** | **P-value** |
| **Costs of re-hospitalization (€),** for PY of follow-up [95% CI] | 13484 [13458; 13509] | 8382 [8370; 8394] | 0.17 [-0.79; 1.12] | 0.7313 |

^a^ Univariate logistic regression with logarithmic transformation (natural logarithm) of the duration of the hospital stay and ICU stay

^b^ Cox model

^c^ Poisson regression model adjusted for duration of follow-up

^d^ Generalized linear regression with gamma distribution and log link

^e^ Generalized linear regression with gamma distribution and log link adjusted for the duration of follow-up

N, number of patients; LT, liver transplantation; SD, standard deviation; CI, confidence intervals; PY, person/year; €, euros
